# Activity Landscape and Molecular Modeling to Explore the SAR of Dual Epigenetic Inhibitors: A Focus on G9a and DNMT1

**DOI:** 10.3390/molecules23123282

**Published:** 2018-12-11

**Authors:** Edgar López-López, Fernando D. Prieto-Martínez, José L. Medina-Franco

**Affiliations:** 1 Medicinal Chemistry Laboratory, University of Veracruz, Agustín de Iturbide Esq. Carmen Serdan, 91700 Veracruz, Mexico; edgar.lopez.593@hotmail.com; 2 Department of Pharmacy, School of Chemistry, National Autonomous University of Mexico, 04510 Mexico City, Mexico; ferdpm4@hotmail.com

**Keywords:** activity cliff, activity landscape plotter, epigenetics, docking, drug discovery, d-tools, molecular dynamics, epi-polypharmacology, SmART, structure-activity relationships

## Abstract

In this work we discuss the insights from activity landscape, docking and molecular dynamics towards the understanding of the structure-activity relationships of dual inhibitors of major epigenetic targets: lysine methyltransferase (G9a) and DNA methyltranferase 1 (DNMT1). The study was based on a novel data set of 50 published compounds with reported experimental activity for both targets. The activity landscape analysis revealed the presence of activity cliffs, e.g., pairs of compounds with high structure similarity but large activity differences. Activity cliffs were further rationalized at the molecular level by means of molecular docking and dynamics simulations that led to the identification of interactions with key residues involved in the dual activity or selectivity with the epigenetic targets.

## 1. Introduction

Technological-scientific advances have allowed to study the molecular machinery involved in the development of chronic diseases, as is the case of neoplastic hematological diseases. There are phenomena related to the development of this type of diseases, e.g., coding mutations, and more recently the deregulation of epigenetic events such as the processes of methylation, acetylation, phosphorylation, etc., which are fundamental in cellular homeostasis. Therefore, there is a need to develop new therapeutic strategies to address abnormal epigenetic modifications.

**Figure 1:**
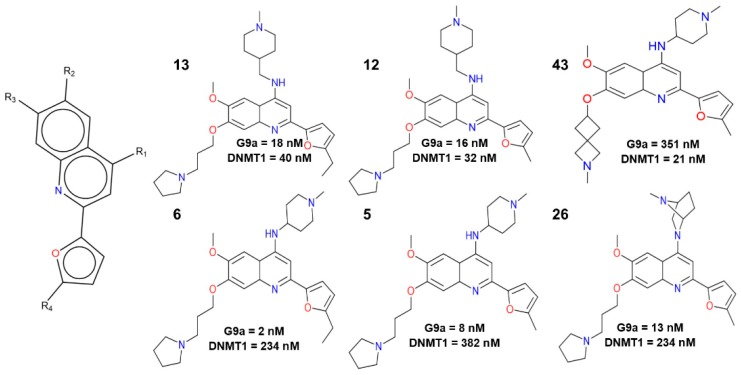
Selected 4-aminoquinoline compounds with the highest activity reported for G9a and DNMT1.

Case in point, the work published by Rabal, et al. serves as a key step forward in this analysis. They synthesized a novel series of 4-aminoquinolines as inhibitors of the enzymes lysine methyltransferase (G9a) and DNA methyltransferase 1 (DNMT1). G9a is an enzyme that methylates the lysine-9 position of histone H3. This action marks the genomic region packaged with these methylated histones for transcriptional repression. DNMT1 is an enzyme that transfers methyl groups to cytosine nucleotides of genomic DNA. This protein is the major enzyme responsible for maintaining methylation patterns following DNA replication. Both epigenetic targets participate collaboratively in the development of neoplastic hematological diseases. Authors of that work carried out in vitro, in vivo and in silico analysis that led to the identification of compounds selective for G9a (**5**, **6** and **26,** Figure 1), selective for DNMT1 (**43**), and dual inhibitors **12** and **13** [1]. Given that the compounds analyzed were obtained from a lead optimization process, the activity landscape modeling for the identification of “dual activity cliffs” and “dual activity switches”, along with their interpretation at the molecular level are crucial to complement the characterization of the structure-activity relationship (SAR) and structure multiple-activity relationships (SmART).

The aim of this work was to better understand the structural modifications of 4-aminoquinoline analogues for dual or selective inhibitory activity of G9a and DNMT1. To this end, single- and dual-activity landscapes were explored, to rapidly uncover activity cliffs and activity switches. The structural features associated with the cliff-forming compounds were further rationalized and explored using molecular docking and dynamics. The significance of this study lies in the identification of structural features that could lead to pharmacophoric hypotheses and eventually develop additional selective and dual inhibitors against the two epigenetic targets. To the best of our knowledge, this work would be the first to characterize the binding site for two epigenetic targets in a parallel protocol. It is expected that the outcome of this study contributes to further advance the epigenetic polypharmacology.

## 2. Results and Discussion

First we present and discuss the results of a qualitative exploration of the SAR of the data sets against two targets, followed by the results of the activity landscape modeling. With the latter leading to the identifications of activity cliffs. A structure-based interpretation of the cliffs was done with docking and molecular dynamics (MD) simulations, which are discussed in the following sub-sections.

### 2.1. Qualitative Analysis of the SAR with SAReport

**Figure 2:**
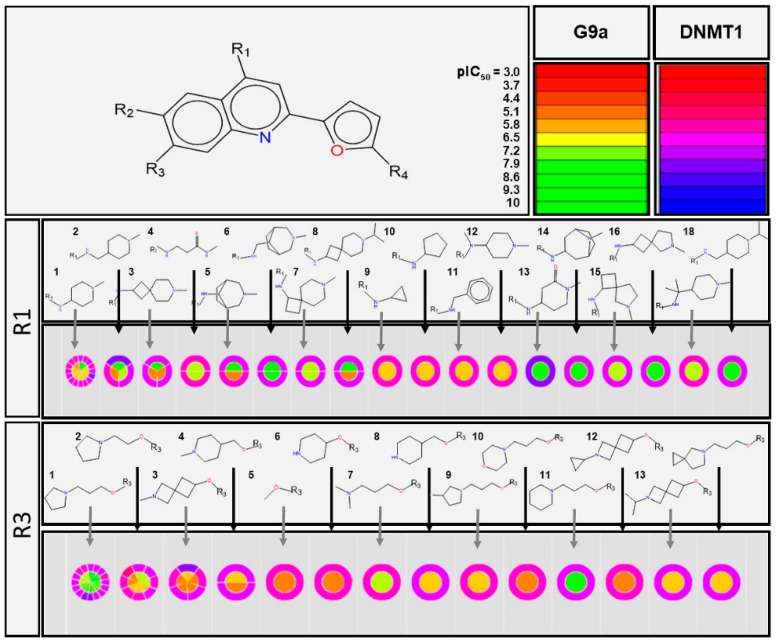
Visual representation of the activity data for compounds with different R_1_ and R_3_ substituents on the dual activity of compounds against G9a and DNMT1. The graph was generated with the SAReport of Molecular Operating Environment.

A qualitative assessment of the SAR was conducted using a data set of 50 compounds, previously tested with G9a and DNMT1 using SAReport a module of Molecular Operating Environment (MOE). Briefly, SAReport (Figure 2) aligns the compounds according to a main core scaffold and visually represent the activity data for each biological target (G9a and DNMT1 in this work) using continuous color scales, e.g., for G9a: from red (low activity) to green (high activity), and for DNMT1: from red (low activity) to blue (high activity) (Figure 2 and Figure S1 in the Supplementary Materials).

Figure 2 shows a visualization of the activity data for the two targets as pie charts: The activity for G9a is represented in the inner part of the circle and that of DNMT1 in the outer ring. When two compounds share the same substituent, the graph is divided into two parts, where each one represents a compound. If there are three compounds sharing a substituent the circular graph will be divided into three parts, and so on. This figure focuses on the variations in activity on G9a and DNMT1 driven by substituents R_1_ and R_3_. The qualitative analysis indicated that the substituents at position R_2_ and R_4_ have a lesser impact on the activity of the set of compounds (Figure S1 in the Supplementary Materials).

Based on the outcome of SAReport, the dual activity of compounds largely increases with the substituents *N*,1-dimethylpiperidin-4-amine (R_1_-2), 1-methyl-4-(methylamino)piperidin-2-one (R_1_-13) and 1-(3-methoxypropyl)pyrrolidine (R_3_-1). The selectivity towards G9a is favored by the substituent 1-(3-methoxypropyl)piperidine (R_3_-11). Whereas for DNMT1, selectivity is mostly driven by the 6-methoxy-2-methyl-2-azaspiro[3.3] heptane (R_3_-3). Thus, overall, the dual activity is driven by the substituents in the position R_1_ and R_3_.

### 2.2. Activity Landscape

#### 2.2.1. SAS Maps

**Figure 3:**
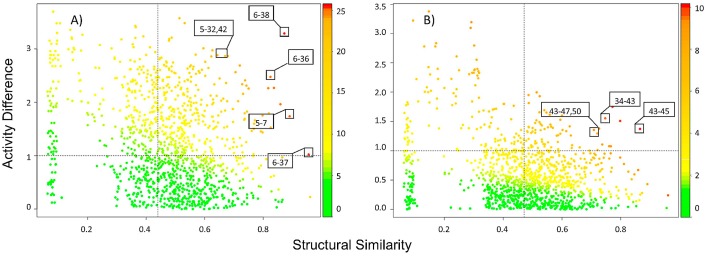
SAS map of compounds with activity against G9a (**A**) and DNMT1 (**B**). Data points are colored by SALI value using a continuous scale from low (green) to high (red).

Figure 3A,B show the Structure-Activity Similarity (SAS) maps for G9a and DNMT1, respectively. Each plot contains 1225 data points, each one representing a pairwise comparison (vide infra). The maps show the relationship between the difference in activity and molecular similarity for each pair of compounds. As detailed in the Materials and Methods section, molecular similarity was measured with the Tanimoto coefficient using MACCS keys fingerprint. Data points are further distinguished by the Structure-Activity Landscape Index (SALI) values, using a continuous color scale from low (green) to high (red), where the activity cliff will have a high SALI value. For both targets, most pairs are colored green and yellow indicating a more continuous SAR: Similar structures with similar activity. This result can be explained, at least in part, because the compounds come from a lead-optimization process [1].

As shown on Figure 3A, a significant proportion of compound pairs have large activity differences (more than one or two log units). This suggests that this set of compounds explores in more detail the SAR for G9a but has a rough activity landscape. However, Figure 3B shows that for DNMT1 most of the pairs have a low activity difference (less than one log unit) and have high structure similarity. This is consistent with a lead-optimization process that is based, in general, in the similarity principle. Thus, the SAR for compounds with DNMT1 activity would be suitable to generate quantitative structure-activity relationship (QSAR) models that overall assume that similar compounds have similar activity [2].

**Figure 4:**
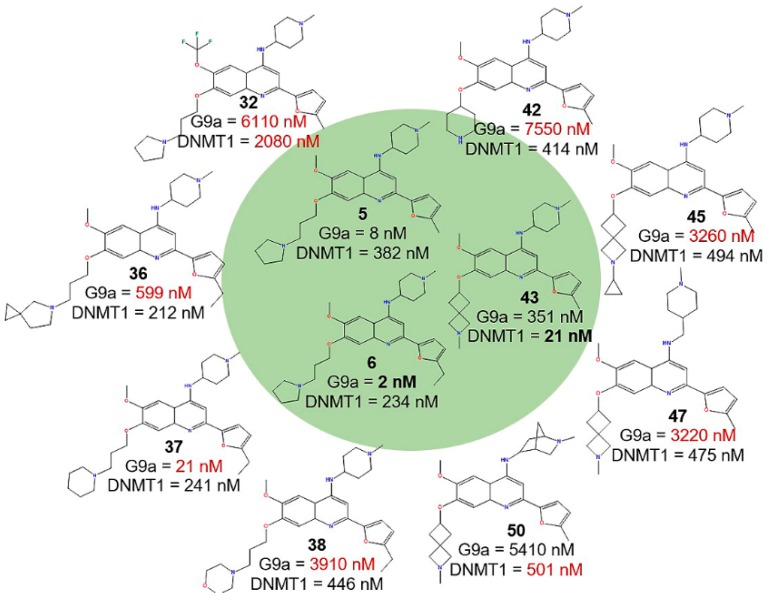
Chemical structures of representative compounds forming activity cliffs.

Specific examples of activity cliffs are labeled in Figure 3A,B. Figure 4 shows the chemical structures of selected activity cliffs. For instance, a prominent activity cliff for G9a is the compound pair **6**-**38** (Figure 3A and Figure 4) that have a small structural change at R_3_. Other examples of compound pairs with small structural changes at R_3_ but large activity differences are **6**-**36**, **6**-**37**, **5**-**32** and **5**-**42**.

A representative activity cliff for DNMT1 is the compound pair **43**-**45** (Figure 3B) that have a small structural change at R_3_. Other examples of activity cliffs with small structural changes at R_1_ are **34**-**43**, **43**-**47** and **43**-**50**.

#### 2.2.2. Quantitative Analysis of SAS Maps

To generate a systematic and quantitative analysis of the activity landscape, the SAS maps were divided into four regions defining thresholds for activity difference and structural similarity (see methods section and Figure 3A). Table 1 summarizes the number of pairs of compounds in the Activity Cliff, Smooth SAR and Similarity Cliff regions identified in the SAS maps generated for G9a and DNMT1, respectively. As stated in the materials and methods section the pair wise similarity was calculated with different fingerprints (MACCS keys, PubChem, and ECFP4).

Results in Table 1 indicate a higher proportion of consensus activity cliffs for G9a (27.8%) vs. DNMT1 (8.9%). This is in line with the previous qualitative analysis of the SAS maps discussed above. However, for both targets, most of the data pairs are located in the Smooth SAR region (33.4 and 61.1%, respectively). This is also consistent with the overall similarity principle.

#### 2.2.3. Activity Cliff Generators

Based on the SAS map and the number of activity cliffs, frequency of compound pairs forming the activity cliff region was quantified. Identifying compounds **5** (7.9%), **6** (9.6%), **12** (9.1%) and **13** (13.4%) as Activity Cliff Generators, i.e., molecules frequently found in the activity cliff region, associated with the largest activity differences [3].

#### 2.2.4. DAD Maps

**Figure 5:**
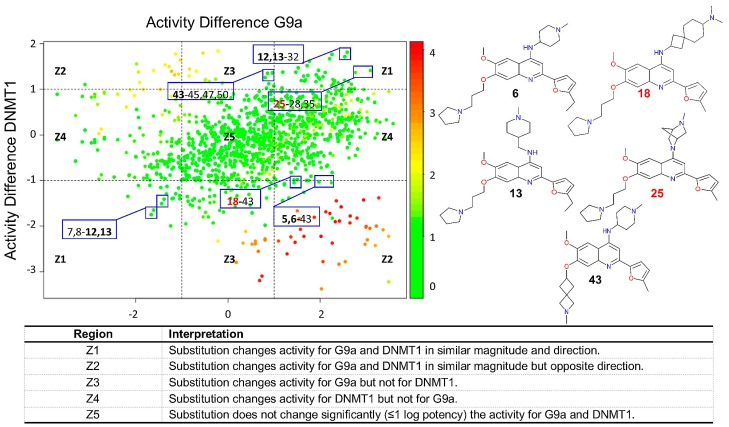
DAD map of compounds with dual activity against G9a and DNMT1. Data points are colored by SALI value. The chemical structures of selected compounds are shown.

Figure 5 shows a Dual-Activity Difference (DAD) map, plotting all pairwise activity differences of the 50 compounds (1225 data points) with G9a (X-axis) and DNMT1 (Y-axis). Therefore, DAD maps facilitate the identification of compounds with selective and dual activity. Figure 5 also provides relevant information of selected pairs of compounds, e.g., pairs with compounds **5** and **6** (selective for G9a), **43** (selective for DNMT1), as well as **12** and **13** (identified as dual compounds) are highlighted. In contrast to the results discussed in previous sections pairs with compound **18** suggest selective activity against G9a. Also, pairs with compound **25** suggest a dual activity.

### 2.3. Molecular Docking

Based on the outcome of the activity landscape, molecular docking was used to generate plausible binding poses with G9a and DNMT1 of selected compounds involved in activity cliffs. Docking was conducted with AutoDock 4 (Molecular Graphics Laboratory, La Jolla, CA, USA), as detailed in the materials and methods section.

**Figure 6:**
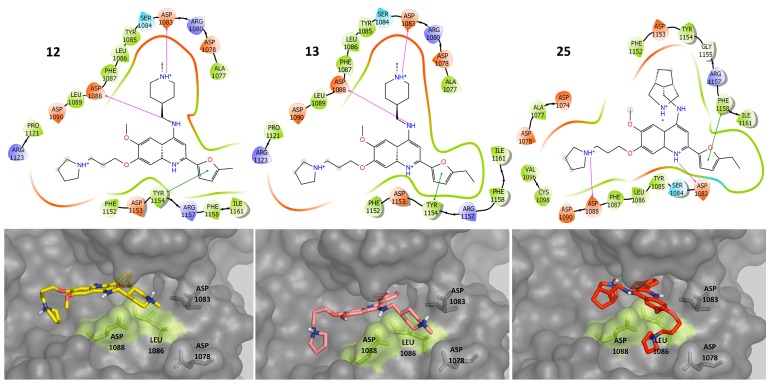
Predicted binding poses of dual compounds against G9a. Key amino acids proposed for the dual activity are highlighted in green.

Figure 6 shows one of the best ranked binding poses with G9a of three representative compounds with dual activity: **12**, **13** and **25**. The three compounds were selected because the DAD map highlighted them as the best compounds with dual activity (Figure 5 and Figure S2). The docking model suggested that the three compounds have protein-ligands contacts with the side chains of ASP 1083, ASP 1088, LEU 1086 and PRO 1121. Also, these compounds make contacts with the backbone of TYR 1154. Alternative binding poses are shown in Figure S3.

**Figure 7:**
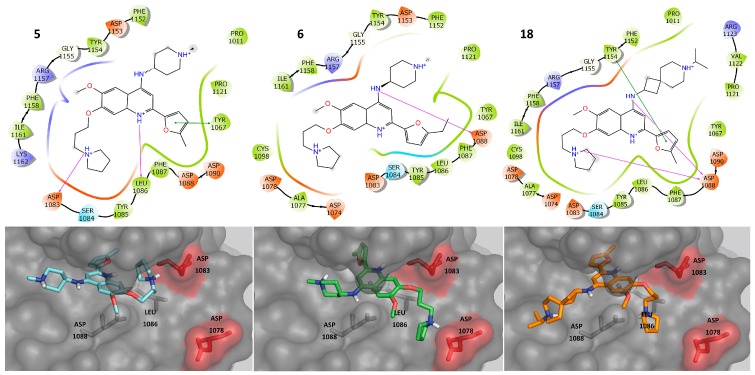
Predicted binding poses of selective compounds against G9a. Key amino acids proposed for the selective activity are highlighted in red.

Figure 7 shows the predicted binding poses of three selected compounds that are selective for G9a: **5**, **6**, **18**. These compounds were selected because the DAD map pointed to these molecules as the best with selective activity against G9a (Figure 5 and Figure S2). These molecules have protein-ligand contacts with the side chains of ASP 1078 and ASP 1083.

We emphasize that the R_3_ substituent (Figure 2) generates electrostatic interactions with polar amino acids in G9a. In addition, the dual compounds make interactions with amino acids inside the pocket (colored green); in contrast, the selective compounds also generate interactions with amino acids on their exterior (colored red). In both cases, the flexibility of the R_3_ substituent is significant in molecular recognition.

**Figure 8:**
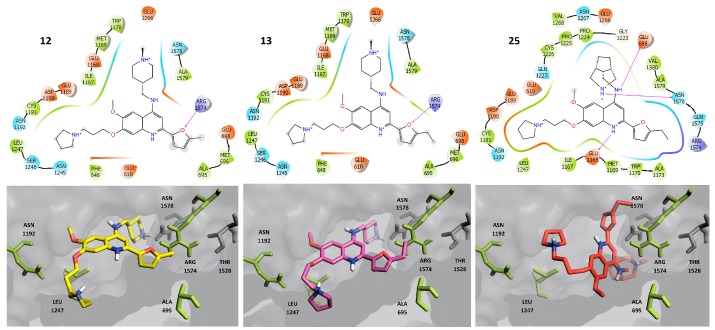
Predicted binding poses of dual compounds against DNMT1. Key amino acids proposed for the dual activity are highlighted in green.

Figure 8 shows the top ranked binding poses of three representative compounds with dual activity against DNMT1: **12**, **13** and **25**. These compounds have preferred protein-ligand contacts with the side chains of ALA 695, ASN 1192, LEU 1247 and ARG 1574.

**Figure 9:**
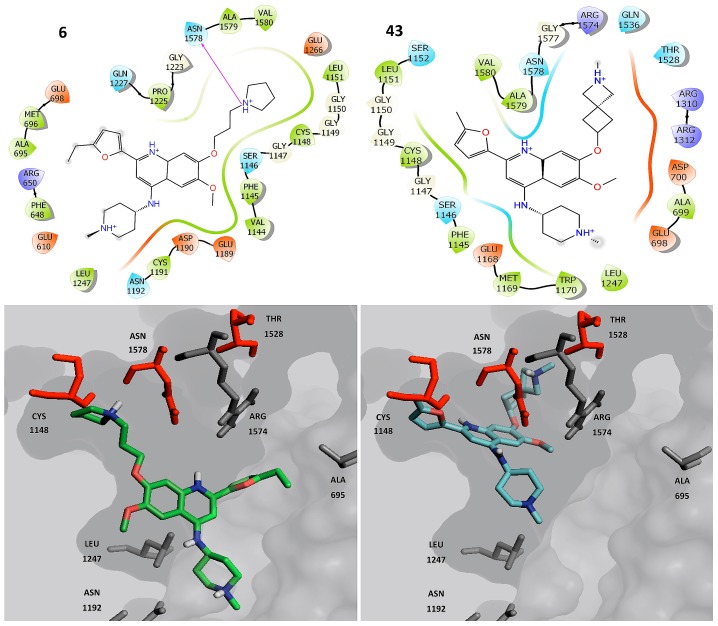
Predicted binding poses of selective compounds against DNMT1. Key amino acids proposed for the selective activity are highlighted in red.

Figure 9 shows the top ranked binding poses of the representative compounds with activity against DNMT1: **43** (this compound was selected because the DAD map refers to them as the best compounds with selective activity against DNMT1, Figure 5 and Figure S2) and **6** (selected because this compound was the starting point to optimize the activity of the whole compound series) [1]. In the predicted binding models, **6** and **43** have protein-ligand contacts with the side chains of THR 1528 and ASN 1578, and contacts with the backbone of CYS 1148.

For DNMT1, the substituents at R_1_ and R_3_ (Figure 2) make electrostatic interactions with polar amino acids close to the surface of the pocket (colored green—Figure 9). In contrast, the selective compounds also make interactions with amino acids distant from the surface (colored red—Figure 9). In both cases, the flexibility of the R_3_ substituent is significant in molecular recognition.

### 2.4. Molecular Dynamics

**Figure 10:**
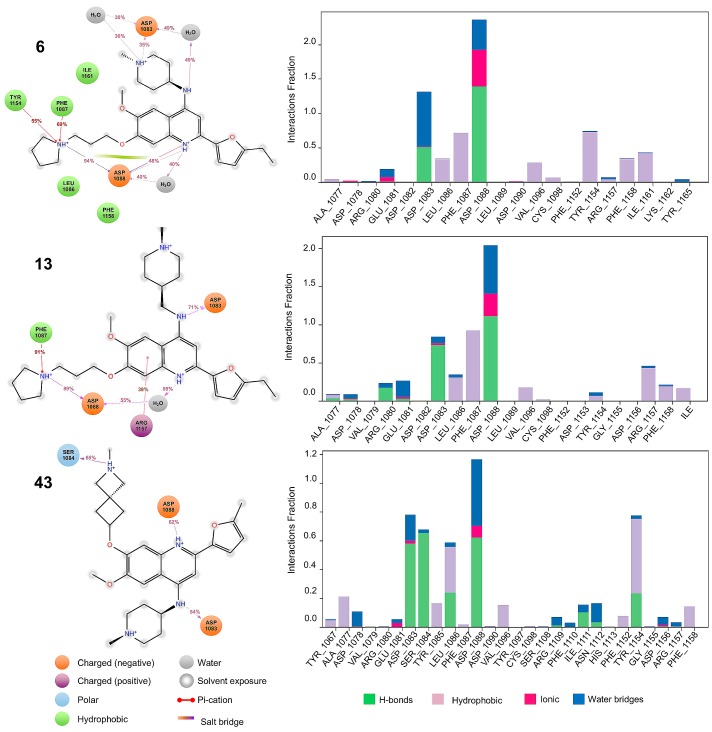
G9a–ligand contact analysis during the 100-ns simulations. The diagrams show the fraction and type of interactions with representative ligands during the simulation.

Based on the insights from activity landscape modeling and docking calculations we performed MD simulations for selected compounds. Figure 10 describes the interactions generated between compounds **6** (selective for G9a), **13** (dual) and **43** (selective for DNMT1) against G9a, for a period of 100 ns under pre-established conditions (see in the materials and methods section). We highlight the hydrophobic and ionic interactions with ASP 1083 and ASP 1088, which are conserved in the selective and dual compounds.

Also, compound **6** (selective for G9a) and **13** (dual) maintain H-bond interactions with ASP 1088 with their respective substituents R_3_. However, the interaction between ASP 1088 is enhanced in compound **6**, where it also presents a “salt bridge” with its scaffold, in contrast to its counterpart **13** (which does not generate it). Other fundamental interactions in the molecular recognition are with PHE 1087 and ARG 1157 that establishes π-cation interactions, respectively. Additionally, the flexibility on R_3_ proved significant, as the dual and selective compounds have increased contacts with the aromatic triad. These observations are further supported by previous evidence on the structural flexibility needed for G9a inhibition [4].

**Figure 11:**
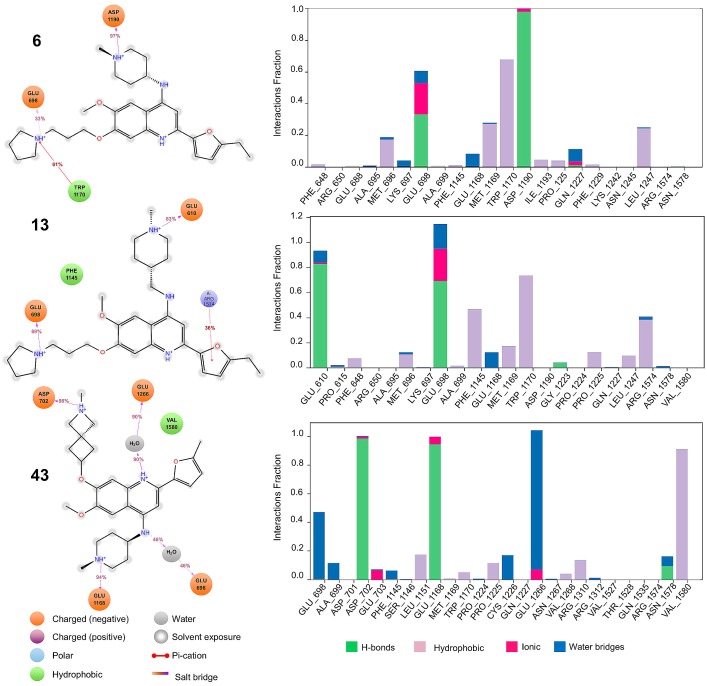
DNMT1–ligand contact analysis during the 100-ns simulations. The diagrams show the fraction and type of interactions with representative ligands during the simulation.

Figure 11 describes the interactions generated between compounds **6** (selective for G9a), **13** (dual) and **43** (selective for DNMT1) against DNMT1. We highlight the hydrophobic and ionic interactions with GLU 698. Likewise, the three compounds maintain hydrophobic and ionic interactions with their respective substituents R_1_ and R_3_.

In addition to the above-mentioned interactions, compound **13** (dual) interacts with ARG 1574 through π-cation interactions, in contrast to its counterparts **6** and **43** (which do not generate it). Finally, another fundamental interaction in dual molecular recognition is with PHE 1145.

## 3. Materials and Methods 

### 3.1. Data Set

This study is based on a novel data set of 50 compounds derived from 4-aminoquinoline with dual activity against G9a and DNMT1 published by Rabal et. al. [1]. All compounds were assayed under the same biological conditions and have IC_50_ values reported for each target. The SMILES representation of the structures and pIC50 (−log IC_50_) values are listed in Table S1 in the Supplementary Materials. For G9a, the pIC_50_ values range from 5.0 to 8.69; For DNMT1 the pIC_50_ values range from 4.3 to 7.67. Overall, the range of activity values for both targets is similar which facilitates the cross-comparisons of the activity landscape (vide supra) [5].

### 3.2. Software and Online Resources

The qualitative SAR analysis was performed with the SAR Report Tool (Chemical Computing Group, Montreal, QC, Canada) implemented in MOE [6]. The activity landscape analysis was carried out with Activity Landscape Plotter, an open web tool (https://www.difacquim.com/d-tools/) that enables the analysis of SAR of screening data sets [7]. This tool facilitates a first and rapid exploration of the SAR of compound data sets with a common scaffold and rapid decomposition of R-groups. The visual representation of the chemical space was done with DataWarrior (Actelion Pharmaceuticals Ltd, Allschwil, Switzerland) [8]. Molecular docking was performed with AutoDock 4 (Molecular Graphics Laboratory, La Jolla, CA, USA), and molecular dynamics studies were done with Desmond (Schrödinger, New York, NY, USA) [9,10].

### 3.3. Activity Landscape

A SAS map is a tool for SAR analysis of compound data sets tested with one molecular target. SAS maps are based on the concept of activity landscape and are suited for the rapid identification of activity cliffs, defined as compounds with a similar high structure but different biological activity [11]. DAD maps allow the analysis of activity landscapes of compound data sets with two biological targets. DAD maps quickly identify “selective switches”, defined as compounds with structural changes that completely invert the selectivity against toe different biological targets [12,13].

SAS and DAD maps are based on systematic pairwise comparisons of the compounds in a data set. Thus, SAS and DAD maps generated in this work represented all 1225 pairwise comparisons between the 50 compounds of the set. Two SAS maps were generated, one for each target, G9a and DNMT1, respectively. In each map the structure similarity was calculated with the ECFP4 fingerprint and the Tanimoto coefficient and was represented on the X-axis. The activity difference was plotted on the Y-axis. In order to differentiate the four major regions in the SAS map two thresholds were set along the X- and Y-axis, respectively. The criteria to select the threshold along the X-axis was the mean of the similarity values of all compounds in the set (0.4712) (calculated with Tanimoto and the ECFP4 fingerprint). The threshold of the activity difference (Y-axis) was set to one logarithmic unit.

The data points in the SAS maps were further colored by their SALI value. This index, as implemented in Activity Landscape Plotter, quantify activity cliffs using the equation proposed by Guha and Van Drie [14,15] in Equation (1):

(1) $$SALI\;i,j=\frac{\left|A_{i}-A_{j}\right|}{1-\mathrm{sim}\left(i,j\right)}$$

where *A_i_* and *A_j_* are the activities of the *i*th and the *j*th molecules, and sim (*i*, *j*) is the similarity coefficient between the two molecules (in this work computed with the ECFP4 fingerprint and the Tanimoto coefficient). The SALI values were mapped onto the SAS maps using a continuous color scale from the structurally most similar pairs (green) to the least similar pairs (red). For the quantitative analysis of SAS maps, the structure similarity was also evaluated with MACCS keys (166-bits) and PubChem fingerprints as implemented in Activity Landscape Plotter [7].

A DAD map was generated plotting on the X- and Y-axis, the absolute value of the activity difference of compounds tested with G9a and DNMT1, respectively. To analyze the DAD maps threshold value of one logarithmic unit were used.

### 3.4. Molecular Docking

#### 3.4.1. Protein Preparation

The crystallographic structures of human G9a (PDB ID: 3RJW) and DNMT1 (PDB ID: 3SWR) were retrieved from the Protein Data Bank (https://www.rcsb.org/) [16,17]. Co-crystal ligands were removed (quinazoline-4-amine CIQ and sinefungin, respectively) were removed. Missing loops and side-chains were added with YASARA [18]. Finally, hydrogen atoms were added, followed by a minimization step with the AMBER99 forcefield in MOE [7].

#### 3.4.2. Ligand Preparation

The ligands were built and energy-minimized in MOE using the MMFF94x force field (Chemical Computing Group, Montreal, QC, Canada). The more stable protomers at physiological pH were identified [19].

#### 3.4.3. Molecular Docking

AutoDock 4 was used to add the solvent model and assign the atomic charges of Gasteiger to proteins and ligands [9]. For G9a, the grid was centered on the carbon atom of the carboxyl group of ASP 1088 (chain A) with a size of 45 × 45 × 45 Å^3^, and for DNMT1 on the carbon atom of the carboxyl group of GLU 1266 (chain A) with a size of 65 × 65 × 65 Å^3^. A grid spacing of 0.375 Å was used. Using the Lamarckian-Genetic algorithm, the binding compounds were subjected to 20 search steps using 2,500,000 energy evaluations, and the default values of the other parameters. The ten best binding poses of the different clusters were generated.

#### 3.4.4. Search for Ideal Conditions

Based on studies that describe the interactions involved in the molecular recognition of G9a and DNMT1. Key residues in the binding pocket for G9a are TYR 1067, ASP 1078, ASP 1074, ASP 1083, ASP 1088, LEU 1086, TYR 1152 and TYR 1154. For DNMT1 the key residues in the binding pocket are SER 1233, MET 1235, TYR 1243, GLU 1269, ARG 1315, ARG 1576 and ASN 1580 [1,20,21]. Compound **6** (not present on the PDBs structures reported) was used to guide the development of a protocol that captured the interactions reported for other active compounds. To this end, different grid sizes were evaluated for G9a (i.e., 20, 30, 40, 45 and 50 Å^3^) and DNMT1 (i.e., 20, 30, 40, 50, 60, 65 and 70 Å^3^). The grid sizes selected were 45 × 45 × 45 Å^3^ for G9a, and 65 × 65 × 65 Å^3^ for DNMT1.

### 3.5. Molecular Dynamics

MD studies of the protein-ligand complexes were performed using Desmond (version 2018-3, Schrödinger, New York, NY, USA) with the OPLS 2005 forcefield [10]. The most representative docking pose for each ligand was used as starting point to initiate the MD simulations. The complexes were prepared with the System Builder Utility in a buffered orthomobic box (10 × 10 × 10 Å), using the transferable intermolecular potential with 3-point model for water (TIP3P). The complexes were neutralized and NaCl was added in a 0.15 M concentration.

Complexes were minimized using the steep-descent (SD) algorithm followed by the Broyden-Fletcher-Goldfarb-Shanno (LBFGS) method in three stages. In the first stage water heavy atoms were restrained with a force constant of 1000 kcal mol^−1^ Å^−2^ for 5000 steps (1000 SD, 4000 LBFGS) with a convergence criterion of 50 kcal mol^−1^ Å^−2^; for the second stage, backbones were constrained with a 10 kcal mol^−1^ Å^−2^ force constant using a convergence criterion of 10 kcal mol^−1^ Å^−2^ for 2000 steps (1000 SD, 1000 LBFGS); and for the third stage the systems were minimized with no restraints for 1000 steps (750 SD, 250 LBFGS) with a convergence criterion of 1 kcal mol^−1^ Å^−2^.

Equilibration was carried out in several steps. Beginning with Brownian Dynamics for 250 ps with the Berendsen thermostat. Followed by simulation on the NVT ensemble, slowly heating from 10 K to 300 K over 3000 ps. At this stage, constraints were enforced on solute heavy atoms, using a constant of 50 kcal/mol.

Finally, equilibration on NPT ensemble used the Berendsen thermostat and Langevin barostat for additional 250 ps. Subsequently, the system was submitted to 130 ns of production runs, under NPT ensemble at 1 bar using the Martyna-Tuckerman-Klein (MTK) barostat and 300 K using the Nose-Hoover thermostat. Electrostatic forces were calculated with the smooth PME method using a 9 Å cut-off, while constraints were enforced with the M-SHAKE algorithm. Integration was done every 1.2 fs, with a recording interval of 50 ps. Finally, the first 30 ns of production runs were removed, this is due to system stabilization after this period [22].

The quality of the simulation and trajectory analyses were carried out with the tools implemented in the Maestro-GUI (Schrödinger, New York, NY, USA) (Figures S4 and S5).

## 4. Conclusions

The concept of epigenetic polypharmacology is increasingly relevant [23,24,25,26]. In parallel, Activity Landscape modeling has allowed the characterization of the epigenetic chemical space [26]. Therefore, this study analyzed and augment the information generated by conventional SAR studies. Activity landscape modeling led to the identification of selective and dual compounds against G9a and DNMT1, which will facilitate the generation of more robust SAR studies for both epigenetic targets. Together, based on the different activity landscapes, the set of compounds is an ideal candidate for the generation of QSAR models against DNMT1 (showing a homogeneous SAR) or for virtual screening against G9a (showing a heterogeneous SAR). From the dual ligand-based drug design it was concluded that the new approaches should consider generating structures derived from 4-aminoquinoline with variations in the substituents R_1_ and R_3_. In addition, it was found that the key interactions between the targets and the selective compounds for G9a and DNMT1 are generated with the substituents R_1_ and R_3_, respectively. From the docking and molecular dynamics studies was confirmed the importance of the interactions with polar amino acids in their respective binding sites. Therefore, ligands (with flexible variations on R_3_ substituent), e.g., compound **13**, shows interaction with these polar amino acids, favoring dual activity. A first perspective of this work is conducting 3D-QSAR studies for the 50 molecules for G9a and DNMT1. Also, a second perspective of the MD simulations is to perform the free energy decomposition to further validate the key residues.

## Figures and Tables

**Table 1:**
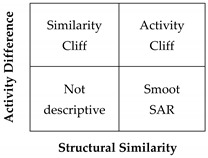
Summary of quantitative analysis of the SAS map for G9a and DNMT1.

Target	Fingerprint	Activity Cliff	Smooth SAR	Similarity Cliff	Not Descriptive
G9a	MACCS	268 (21.8%)	320 (26.2%)	290 (23.7%)	347 (28.3%)
	PubChem	432 (35.3%)	468 (38.2%)	142 (11.6%)	183 (14.9%)
	ECFP4	324 (26.5%)	439 (35.8%)	171 (13.9%)	291 (23.8%)
	Consensus *	**341 (27.8%)**	**409 (33.4%)**	**201 (16.4%)**	274 (22.4%)
DNMT1	MACCS	55 (4.5%)	532 (43.5%)	488 (39.9%)	147 (12.1%)
	PubChem	92 (7.6%)	755 (61.9%)	262 (21.5%)	110 (9.0%)
	ECFP4	177 (14.5%)	949 (77.6%)	71 (5.8%)	25 (2.1%)
	Consensus *	**108 (8.86%)**	**745 (61.1%)**	**274 (22.4%)**	94 (7.73%)

* Consensus calculated based on the average of existing compounds in each region of the SAS map according to different fingerprints.

## Supplementary Materials

The following are available online, Table S1: Compound data set, Figure S1: Visual representation of the activity data for compounds with different R_2_ and R_4_ substituents on the dual activity of compounds against G9a and DNMT1, Figure S2: Gini Index Plot of compounds with selective and dual activity against G9a and DNMT1, Figure S3: Alternative binding poses of the dual compounds against G9a and DNMT1, Figure S4: RMSD and RMSF of G9a during the MD, Figure S5: RMSD and RMSF of DNMT1 during the MD.
