# Supplementary figures and images for: Activity Landscape and Molecular Modeling to Explore the SAR of Dual Epigenetic Inhibitors: A Focus on G9a and DNMT1
Source: Molecules. 2018 Dec 11;23(12):3282. doi: 10.3390/molecules23123282 (PMC6321328; doi:10.3390/molecules23123282)

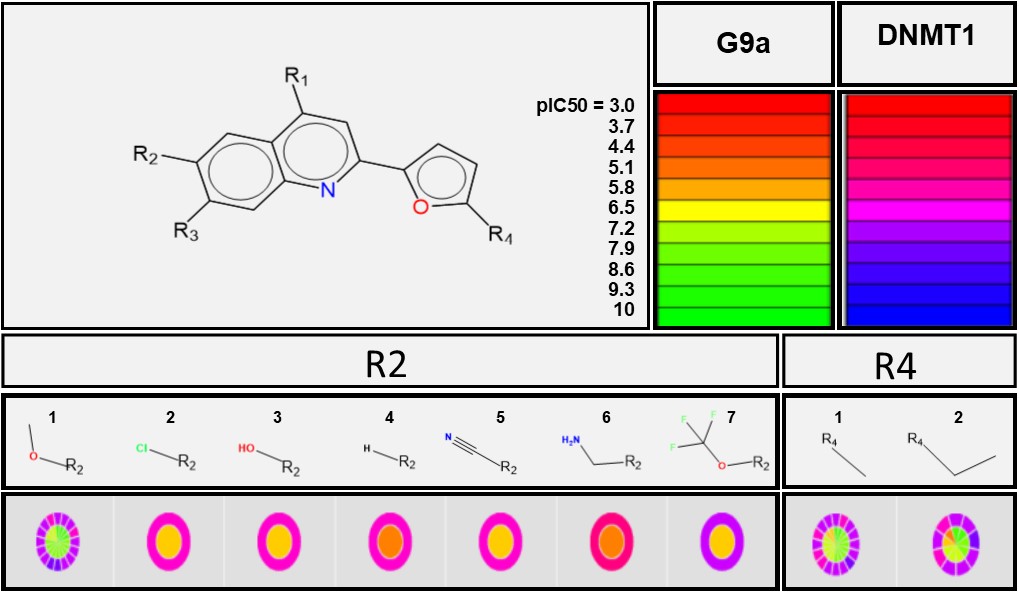

Supplement: Supplementary file 1 [file molecules-23-03282-s001.zip › Figure_S1.jpg]

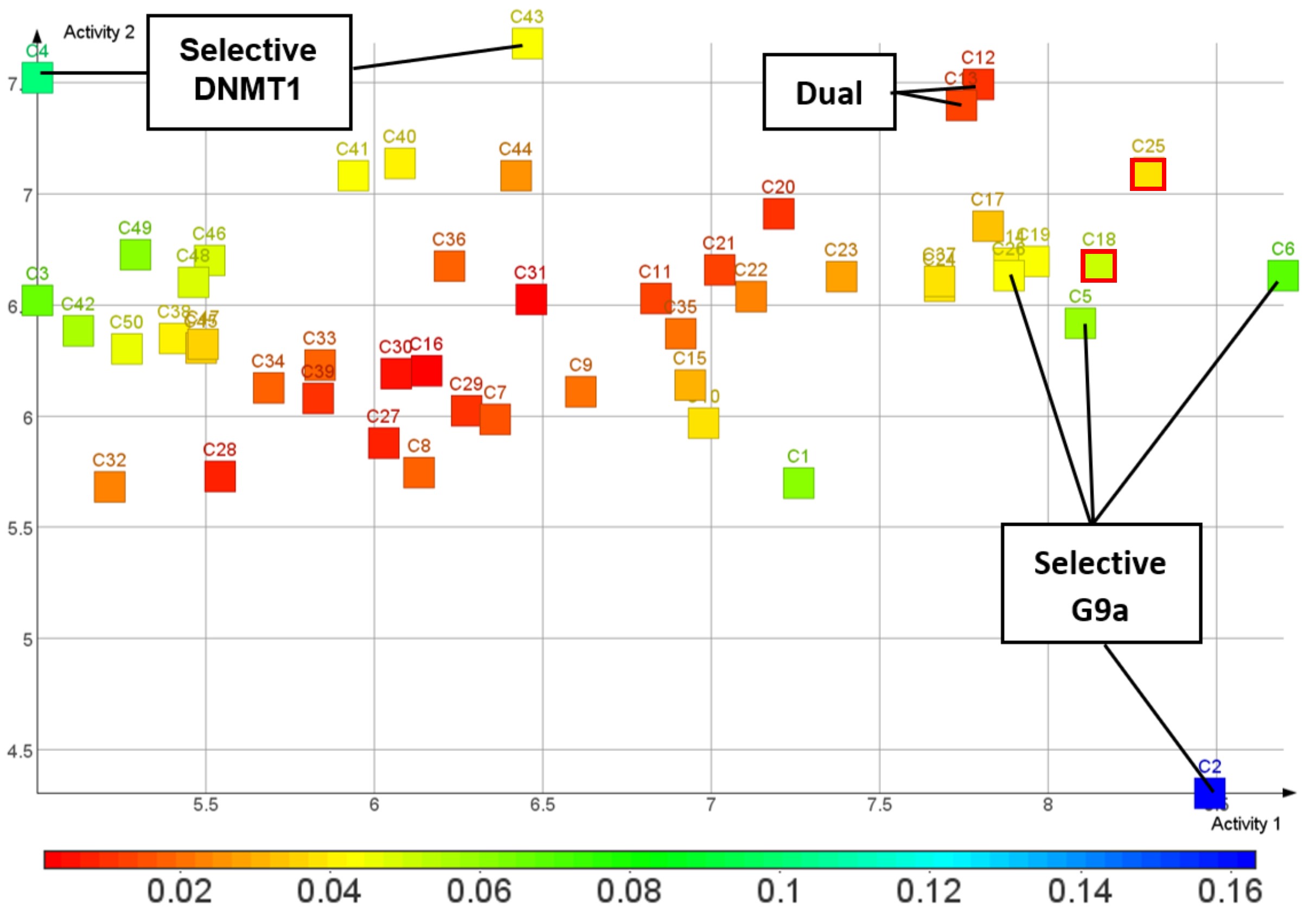

Supplement: Supplementary file 1 [file molecules-23-03282-s001.zip › Figure_S2.jpg]

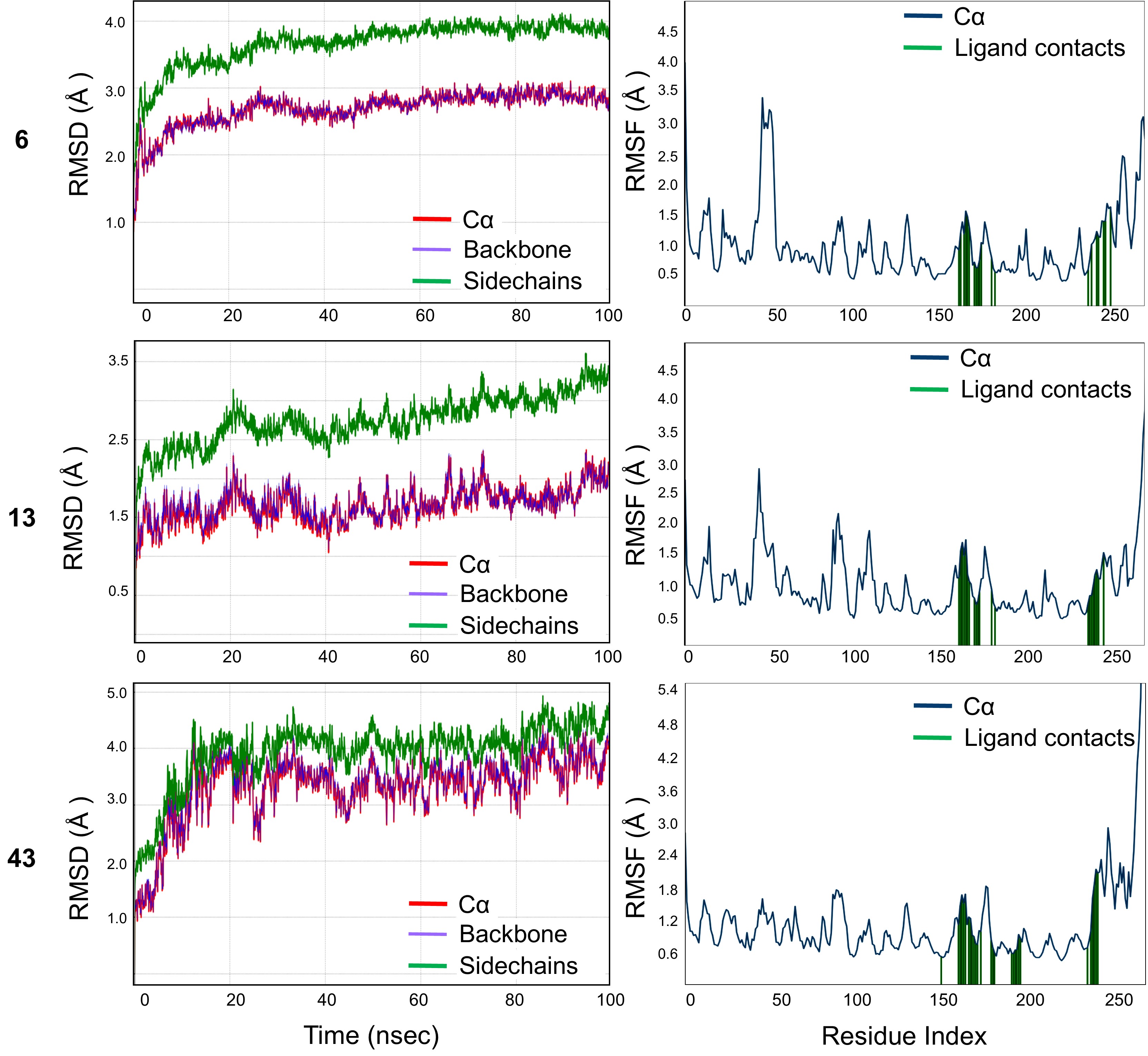

Supplement: Supplementary file 1 [file molecules-23-03282-s001.zip › Figure_S4.jpg]

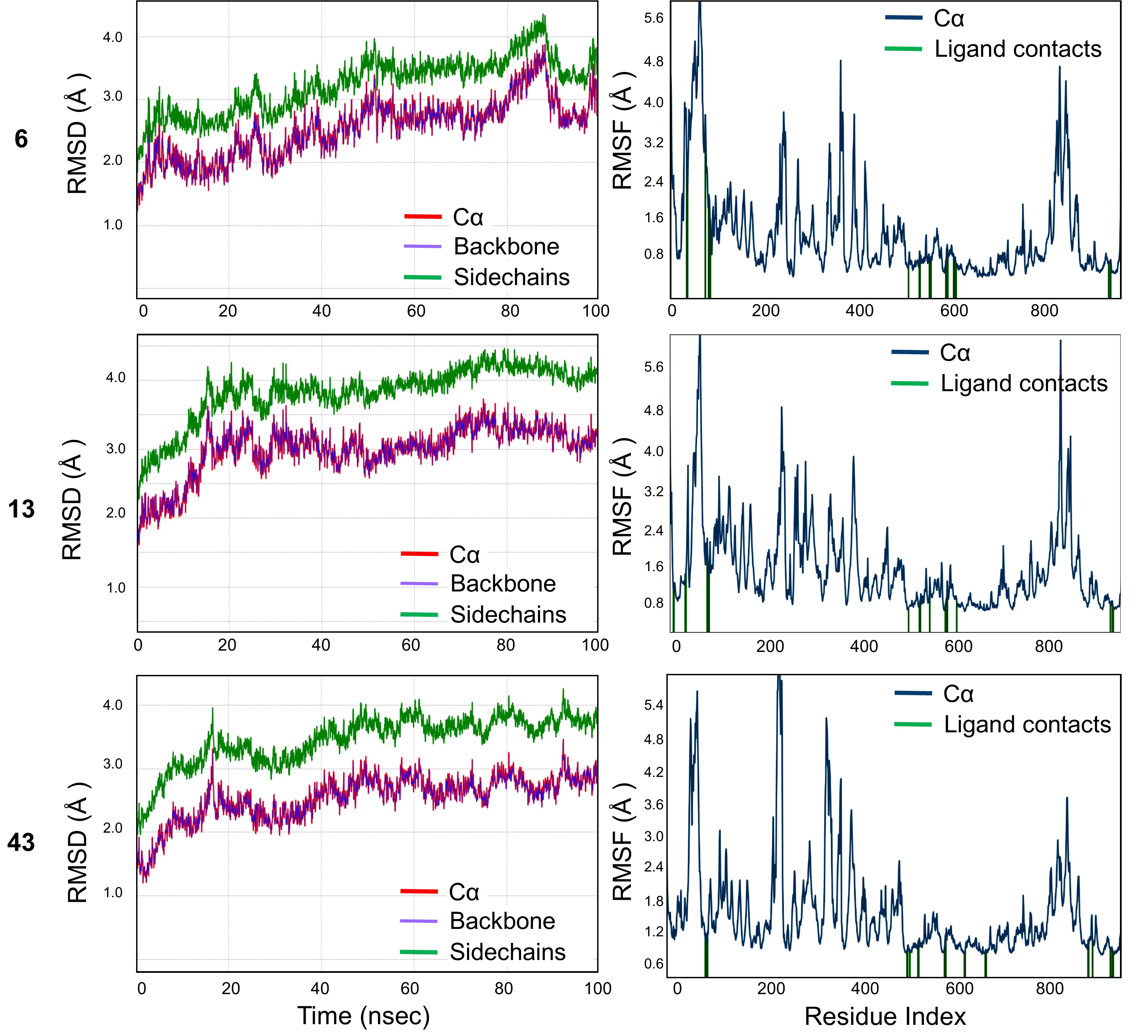

Supplement: Supplementary file 1 [file molecules-23-03282-s001.zip › Figure_S5.jpg]

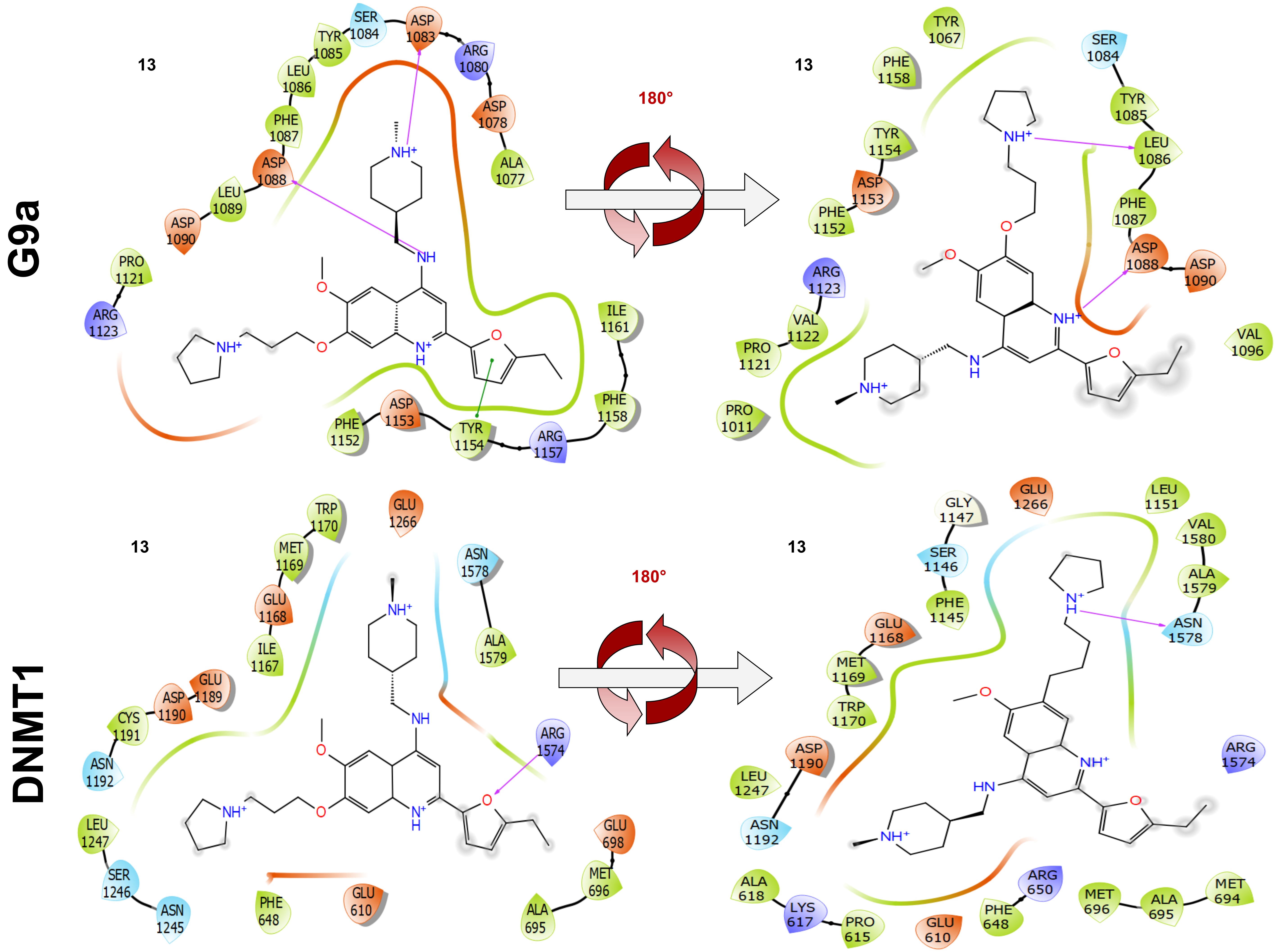

Supplement: Supplementary file 1 [file molecules-23-03282-s001.zip › Figure_S3.jpg]
